# The neural basis of belief-attribution across the lifespan: False-belief reasoning and the N400 effect

**DOI:** 10.1016/j.cortex.2020.01.016

**Published:** 2020-05

**Authors:** Elisabeth E.F. Bradford, Victoria E.A. Brunsdon, Heather J. Ferguson

**Affiliations:** a University of Kent, UK; b University of Dundee, UK

**Keywords:** Social cognition, False beliefs, Belief-attribution, Event-related brain potentials, Ageing

## Abstract

The current study examined how social cognition – specifically, belief-state processing – changes across the lifespan, using a large sample (*N* = 309) of participants aged 10–86 years. Participants completed an event-related brain potential study in which they listened to stories involving a character who held either a true- or false-belief about the location of an object, and then acted in a manner consistent or inconsistent to this belief-state. Analysis of the N400 revealed that when the character held a true-belief, inconsistent outcomes led to a more negative-going N400 waveform than consistent outcomes. In contrast, when the character held a false-belief, *consistent* outcomes led to a more negative-going N400 waveform than inconsistent outcomes, indicating that participants interpreted the character's actions according to their own more complete knowledge of reality. Importantly, this egocentric bias was not modulated by age in an early time window (200–400 msec post-stimulus onset), meaning that initial processing is grounded in reality, irrespective of age. However, this egocentric effect was correlated with age in a later time window (400–600 msec post-stimulus onset), as older adults continued to consider the story events according to their own knowledge of reality, but younger participants had now switched to accommodate the character's perspective. In a final 600–1000 msec time window, this age modulation was no longer present. Interestingly, results suggested that this extended egocentric processing in older adults was not the result of domain-general cognitive declines, as no significant relationship was found with executive functioning (inhibitory control and working memory).

## 1 Introduction

The ability to understand other people's mental states – in terms of their motivations, beliefs, desires, and knowledge states – plays a critical role in daily life, allowing successful interactions to occur. These ‘Theory of Mind’ (ToM) abilities are key predictors of successful social outcomes; individuals who report difficulties in their ToM abilities often report impairments in social interactions, both in clinical populations (e.g., Baron-Cohen et al., 1985, Couture et al., 2006, Frith, 2001), and also in typical human aging (e.g., Bailey et al., 2008, Moran, 2013, Sullivan and Ruffman, 2004). Whilst there has been much empirical work on social cognition abilities, it remains unclear *how* these social cognition capacities are utilized in our daily lives, and, importantly, the extent to which these abilities change or remain stable across the lifespan. The current study examined the electrophysiological basis of tracking other peoples' (false) beliefs and integrating events according to those beliefs (i.e., understanding events from another person's perspective). Specifically, we tested whether and how belief-attribution capacities change across the lifespan in a large sample (*N* = 309) of participants aged 10–86 years old, and examined whether these changes reflect domain-general declines in cognitive skills (e.g., executive functions), versus domain-specific changes in social cognition.

Recent research has shown that the social brain continues to develop throughout adolescence (Blakemore and Mills, 2014, Dumontheil, 2016), and that these structural changes underlie major developmental progressions in social cognition, which interact with improvements in cognitive control (Humphrey and Dumontheil, 2016, Mills et al., 2015). At the other end of the age spectrum, older adults experience greater difficulties in engaging ToM abilities compared to younger adults (Bailey and Henry, 2009, Catelli et al., 2010, German and Hehman, 2006, McKinnon and Moscovitch, 2007). For instance, older adults have been shown to be impaired compared to younger adults in their ability to recognize emotions (Grainger, Henry, Phillips, Vanman, & Allen, 2015), attribute mental states to story characters (Maylor, Moulson, Muncer, & Taylor, 2002), and switch from their own to someone else's perspective (Martin et al., 2019). The social consequences of difficulties with ToM can be severe, limiting social-communicative abilities, which in turn can lead to feelings of loneliness and depression, particularly in later life (e.g., Bailey et al., 2008, Sullivan and Ruffman, 2004). Further, it has been shown that ToM abilities predict social functioning outcomes across the neurotypical human lifespan, highlighting the importance of furthering our understanding of how ToM changes and develops across the lifespan Apperly, Samson, & Humphreys, 2009, Dumontheil, Apperly, & Blakemore, 2010, Schneider et al., 2017. Whilst prior studies have aimed to localise the underlying mechanisms of ToM capacities using neuroimaging or source localisation methods (e.g., Gallager and Frith, 2003, Kang et al., 2018, Vogeley et al., 2001), it remains largely unknown how these social cognitive mechanisms may change across the healthy lifespan, and how this may be reflected in temporally sensitive neuroimaging measures, such as event-related potentials. Results ascertained from healthy, neurotypical adults can significantly further our understanding of ToM processes, including advancing our understanding of the neural changes associated with ToM processes across the lifespan, and how social changes during different stages of life may impact – or be impacted by – ToM engagement.

Apperly and Butterfill (2009) proposed a distinction between explicit and implicit ToM, where implicit ToM refers to automatic tracking of an agent's perspective, and explicit ToM refers to deliberate consideration of an agent's mental states. In other words, explicit ToM involves an individual purposefully and deliberately reasoning about others' beliefs, whereas implicit ToM, which is argued to exist alongside the explicit mental state tracking system, involves processing of another person's mind without a conscious, explicit intention to do so (e.g., Schneider et al., 2017; Back & Apperly, 2010). Neuroimaging research has revealed substantial overlap in brain areas involved in implicit and explicit ToM (e.g., Naughtin et al., 2017), though differences in performance have been observed in these two domains. When examining explicit ToM abilities, tasks tend to require responses that demonstrate an overall understanding of different ToM scenarios (e.g., yes/no answers; explicit verbal/pointing responses). Prior studies have shown that older adults perform consistently worse than younger adults on these explicit measures, indicating a decline in explicit ToM abilities with advancing age (see Henry, Phillips, Ruffman, & Bailey, 2013 for a meta-analysis). More recently however, research has begun to use more sensitive measures, such as eye-tracking, response times, and electrophysiological methods (e.g., event-related potentials, ERPs), to examine the implicit cognitive processes underlying ToM engagement in adults (e.g., Ferguson et al., 2017, Ferguson et al., 2010, Schneider et al., 2012). This research has demonstrated that, despite declines in older age seen in explicit ToM abilities, some aspects of *implicit* ToM processing may remain intact in older age. For instance, Grainger, Henry, Naughtin, Comino, and Dux (2018) conducted a study in which younger and older adults watched a series of videos where true- or false-belief states of characters were manipulated, whilst eye movements were recorded. Analysis of anticipatory looking behaviour revealed similar success levels for implicit false-belief processing in the two groups, despite older adults showing a deficit compared to the younger adults in explicit ToM processing abilities (assessed using an emotion-recognition task).

This distinction between implicit and explicit ToM task performance is further demonstrated by developmental research that has shown advanced ToM skills are present in explicit measures from around 5–7 years old (Baron-Cohen et al., 1997, Brent et al., 2004, Wellman et al., 2001), but that even healthy adults make errors when judging the perspective of another person, suggesting a resilient egocentric bias/‘pull of reality’ in processing of mental states during ToM use (Birch and Bloom, 2007, Bradford et al., 2018, Ferguson, Apperly and Ahmad et al., 2015, Ferguson and Breheny, 2011, Ferguson and Breheny, 2012, Keysar et al., 2000). However, other research has suggested that, at least at an implicit level, another person's perspective is automatically processed (e.g., Kovács et al., 2010, Samson et al., 2010, Schneider et al., 2017). For example, Surtees and Apperly (2012) found that, in a visual perspective taking task in which an avatar could see either the same or a different number of dots as the participant in a virtual room, both children and adults were slower to respond when the avatar's view differed from the participant's, even when this was irrelevant to the task (i.e., when asked to respond from the ‘self’ perspective, so that the avatar's perspective could be ignored). These results were consistent with findings from Samson et al. (2010) and Qureshi, Apperly, and Samson (2010) who report that participants' judgments about their own perspectives were both slower and more error prone when an avatar in the task had a different perspective. This research indicates a differentiation between implicit ToM – where the mere presence of a social agent can slow down an individual's responses, indicating automatic processing of other people's perspectives – and explicit ToM, where adults are shown to make egocentric errors, suggesting a failure to engage in ToM abilities.

Alongside eye-tracking measures, recent research has also utilized ERPs to examine the neural substrates of ToM processing, either using tasks that require participants to make an explicit judgment about a belief-state of a story character (e.g., Liu et al., 2004, Sabbagh and Taylor, 2000, Zhang et al., 2009) or tasks that require participants to observe pictorial depictions of different belief-states, providing a more implicit measure of ToM processing (e.g., Kühn-Popp et al., 2013, Meinhardt et al., 2012). When participants were explicitly prompted to make belief inferences this elicited a frontally distributed late slow wave (LSW) (Liu et al., 2004, Sabbagh and Taylor, 2000), which was similar to the more widely distributed LSW that was elicited when belief inferences were implicit. These overlapping brain responses indicate that people continue to consider other's beliefs, even when they were not directly prompted to track the other person's mental state. Further ERP evidence for implicit ToM processing has been found among young adults by examining modulations of the N400 effect. The N400 is a centro-parietal component that is sensitive to stimulus predictability and semantic integration processes during language comprehension, showing more negative-going amplitudes when an anomaly is detected (Kutas and Hillyard, 1980, Lau et al., 2013, Nieuwland et al., 2018, Westley et al., 2017).

In Ferguson, Cane, Douchkov, and Wright (2015)'s study, participants read a series of short narratives in which a character held a true or false belief about the location of an object, and subsequently acted in a manner that was consistent or inconsistent with this belief state. ERPs were time-locked to the visual onset of the sentence-final critical word (i.e., the object's location). For instance, if a character witnessed an object being moved (true-belief) from Location A to Location B, the character should look for the object in Location B (consistent outcome, where both the character and they know it to be located), rather than Location A (inconsistent outcome). In contrast, if the character did not witness the object being moved (false-belief), the character should look for the object in the original Location A (consistent outcome, even though the object is no longer there) and not Location B (inconsistent outcome, even though this is the true object location). Results showed a classic N400 effect in true-belief scenarios, whereby inconsistent actions elicited a more negative-going N400 than consistent actions. However, this pattern was reversed for false-belief scenarios, as consistent trials elicited a more negative-going N400 than inconsistent trials. This indicated that, when reading the stories, participants were not automatically integrating events according to the character's beliefs, but rather were processing the stories from their own egocentric perspective (i.e., their own knowledge of reality).

Interestingly, contrasting effects have been found among adolescent participants (aged 10–15 years) who completed a similar reading paradigm, while another person was either present or absent in the room (Westley et al., 2017). In Westley et al.’s study, adolescent participants read a story presented on a computer screen in the presence of a confederate. The start of each story was presented to just the adolescent, with the confederate in the room but unable to see the computer screen, creating different knowledge states for the participant and the confederate. The final sentence of the story presented either a plausible or implausible outcome, and was seen by both the participant and the confederate. Results of this joint comprehension task showed a significant N400 effect (i.e., more negative-going N400 effect following implausible outcomes *vs* plausible sentence outcomes) when the final sentence was implausible for the confederate who had not read the full story, even though it was plausible for the fully informed participant. These results suggest that the adolescent participants were automatically simulating the perspective of the other person during the task, and considered this more limited perspective when integrating events in the stories, even though events in the story made sense from their own perspective. Though there are clear methodological differences between Ferguson, Apperly and Ahmad et al., 2015, Ferguson, Cane and Douchkov et al., 2015 and Westley et al.’s studies (in terms of language content, and social environment), it is puzzling that adolescents should have *better* awareness of others' perspectives compared to adults. These mixed results therefore highlight the importance of examining implicit mental state attribution capacities across the lifespan in a single task, which will allow novel insights into how these abilities and underlying neural mechanisms may change at different ages.

Understanding of the processes underlying implicit mental state attribution is important, allowing further insight into potential underlying causes of age-related difficulties in ToM, which in turn can have important functional outcomes (Grainger et al., 2018, Henry et al., 2016). Critically, it is important to further our understanding of how these processes may change across normal aging, including during adolescence, and younger, middle, and older age, as well as building on our knowledge of what may be driving these changes. For instance, there is significant evidence of a link between ToM and executive functioning abilities, particularly inhibitory control (Bradford et al., 2015, Carlson and Moses, 2001, Perner and Lang, 2000) and working memory (Cane et al., 2017, Carlson et al., 2002, Davis and Pratt, 1996, Lin et al., 2010). These links make sense given that successful social cognition requires one to hold in mind multiple perspectives (i.e., working memory), and suppress irrelevant perspectives (i.e., inhibitory control). Moreover, there is now robust evidence that these cognitive abilities decline with advancing age (Elderkin-Thompson et al., 2008, Salthouse et al., 2003), with emerging evidence suggesting that some aspects of this age-related cognitive decline are manifest from 20–30 years old, and decreases at a faster rate with increasing age (Klindt et al., 2017, Salthouse, 2009, Singh-Manoux et al., 2012). Taken together, these findings raise the question of whether changes in ToM abilities (such as increases in egocentric bias during ToM engagement) are driven by domain-general cognitive factors, such as executive function abilities, or whether they are specifically related to changes in social cognition abilities?

The current study adapted the false-belief ERP task in Ferguson, Apperly, et al. (2015) and Ferguson, Cane, et al. (2015) to the auditory domain, examining the N400 as a marker of belief integration, in an age sample of participants spanning adolescence (10 years) to older age (86 years). We examined effects over early and late phases of the N400 (200–400 msec and 400–600 msec), based on previous observations that auditory N400s tend to begin earlier and last longer than N400s elicited in the visual modality (Kutas and Federmeier, 2011, Kutas and Van Petten, 1994), as well as in a later ‘wrap-up’ period (600–100 msec). Moreover, this early/late analysis of the N400 allowed us to examine whether an age-related temporal delay exists in narrative-based ToM processing, following research showing that typical N400 semantic congruity effects at the scalp gets smaller, slower and more variable with age (Kutas & Iragui, 1998). Participants also completed measures of inhibitory control and working memory, to provide measures of general cognitive skills, allowing analysis of whether any age-related changes in ToM abilities are due to declines in *social* skills per se, or whether they are due to general changes in cognitive domains. By including both adolescents – a period of rapid, significant cognitive and social development – as well as middle-aged and older individuals, the current study includes groups of participants that have, to-date, not been at the forefront of research into ToM processes, allowing us to further our understanding of how and *when* implicit mental state attribution processes change across the lifespan.

In line with the study aims, there were three key predictions: First, we expected to replicate Ferguson, Apperly and Ahmad et al., 2015, Ferguson, Cane and Douchkov et al., 2015 findings in a large sample of participants, and in the auditory modality. That is, a typical N400 effect was expected in true-belief conditions, but a reversed N400 effect was expected in false-belief conditions. Second, if social cognitive skills undergo general declines with advancing age, then we predicted that this reversed N400 effect in false-belief conditions would be larger with increasing age. Alternatively, if implicit social skills remain intact in older age, we would not expect age to correlate with N400 effects in false-belief trials, indicating that all participants experience the same degree of egocentric interference on these trials. Finally, we expected to see cognitive skills (i.e., working memory and inhibitory control) declining with advancing age, and it was predicted that these capacities would correlate with the consistency effects in false-belief story conditions.

## 2 Method

We report how we determined our sample size, all data exclusions (if any), all inclusion/exclusion criteria, whether inclusion/exclusion criteria were established prior to data analysis, all manipulations, and all measures in the study.

### 2.1 Participants

A total of 339 participants (112 males) took part in this study as part of a larger task battery. Of this full sample, 20 participants did not have EEG data (due to computer difficulties or participant declining to complete the task), and a further 10 participants were excluded from analysis due to noisy EEG recordings (retaining less than 60% segments in one or more of the critical test conditions after pre-processing). This resulted in a final sample of 309 participants (101 males), aged 10–86 years. Participant details, including IQ (assessed using the Wechsler Abbreviated Scale of Intelligence – Second Edition), are presented in Table 1, divided into four age groups for illustrative purposes. To calculate socio-economic status (SES), participants (if aged over 18) or parents of participants (if aged under 18) reported on their level of education, household income, and their occupation (job title and industry). To calculate an SES index, education level was coded on a scale of 1–6 (from No qualifications – Postgraduate Degree), and household income and occupational class were coded on a scale of 1–7. These three scores were summed to derive an SES index, with lower scores indicating lower SES.

**Table 1:** Participant Characteristics (mean values, with SDs in parenthesis).

	All	Adolescents (10–19 yrs)	Young Adults (20–39 yrs)	Middle Adults (40–60 yrs)	Older Adults (61–86 yrs)
*N*	309	66	69	86	88
Gender (f:m)	208:101	34:32	49:20	63:23	62:26
Age (years)	43.83 (22.05)	14.39 (3.01)	29.25 (6.26)	50.60 (6.24)	70.72 (6.62)
Verbal IQ	109.83 (12.26)	106.42 (10.28)	105.77 (9.33)	106.28 (11.71)	119.06 (11.45)
Perceptual Reasoning IQ	108.74 (12.78)	107.62 (13.30)	106.71 (11.30)	105.29 (12.91)	114.56 (11.54)
Full Scale IQ	110.53 (12.22)	107.91 (10.78)	107.03 (9.36)	106.56 (12.23)	119.14 (11.05)
SES Index	13.75 (3.65)	13.70 (3.99)	13.31 (4.01)	14.13 (3.63)	13.76 (3.13)

All participants were native English speakers, had normal or corrected-to-normal vision, had no known neurological disorders, and no mental health or autism spectrum disorder diagnoses. Participants were recruited from a community sample in the local area of Kent, U.K., using a variety of recruitment strategies (e.g., newspaper adverts, local groups, word-of-mouth, Kent Child Development Unit). Sample size was determined a-priori during grant application, allowing a large data sample across the lifespan to be collected. The Ethical Committee of the School of Psychology, University of Kent, U.K., approved the study.

### 2.2 Materials and design

The tasks described below were programmed using E-Prime software.

#### 2.2.1 False-belief ERP task

The false-belief ERP task was modified from Ferguson, Apperly, et al. (2015) and Ferguson, Cane, et al. (2015). Here, verbal stimuli were presented to participants auditorily through headphones, rather than visually, which allowed us to control for different reading times among the different aged participants. The task involved 120 experimental items (see Table 2 for examples; full experimental stimuli are available at: https://osf.io/pw7h6/ https://osf.io/pw7h6/). Each item was constructed of three sentences, depicting classic change of location true/false belief scenarios. The first sentence introduced a character and described the character placing a target object in a specific location. The second sentence then described a second character moving the target object to a new location. Importantly, this action was either observed (resulting in a true-belief about the target object's location) or not observed (i.e., not seen, resulting in a false-belief about the target object's location) by the first character. The third, final, sentence described the first character looking for the target object, referring to them looking in a location that was either consistent or inconsistent with their true/false belief state. This resulted in a 2 (belief: true *vs.* false) × 2 (consistency: consistent *vs.* inconsistent) within-subjects design. There were four presentation lists of experimental stimuli, with each list containing 120 experimental items, 30 in each of the four conditions, using a Latin square design. In addition, there were 65 unrelated filler stories (approximately the same length as experimental items, but did not depict beliefs) that were randomly interspersed among the experimental items. Participants were randomly assigned to read one of the four test lists, meaning that each participant heard each target sentence in one of the four conditions (i.e., they did not hear the same story more than once).

**Table 2:** Example experimental item in each of the four test conditions. Target word is underlined here for illustration.

True Belief	Consistent	Nick bought some chocolate and put it in the cupboard. Later, Nick noticed Emily move the chocolate to the fridge. When Nick wanted to eat some chocolate he looked in the fridge.
	Inconsistent	Nick bought some chocolate and put it in the cupboard. Later, Nick noticed Emily move the chocolate to the fridge. When Nick wanted to eat some chocolate he looked in the cupboard.
False Belief	Consistent	Nick bought some chocolate and put it in the cupboard. While Nick was out for a run, Emily moved the chocolate to the fridge. When Nick wanted to eat some chocolate he looked in the cupboard.
	Inconsistent	Nick bought some chocolate and put it in the cupboard. While Nick was out for a run, Emily moved the chocolate to the fridge. When Nick wanted to eat some chocolate he looked in the fridge.

In the task, each trial began with the presentation of a single centrally located red fixation cross shown for 500 msec to signal the start of a new trial. After this time, the fixation cross turned black (remaining on screen for the duration of the trial), and the audio stimuli began following a 500 msec delay. Target words were always the final word of the third sentence. Event related potentials for analysis were time-locked to the auditory onset of the target word. After each sentence, there was a blank screen for 1000 msec before the next trial began. One third of the experimental stories (i.e., 10 in each condition) and 27 of the filler stories were followed by a question about how appropriate the target character's actions were, and participants were prompted to respond using a Likert-Type scale from 1 = very appropriate to 5 = very inappropriate. These questions were included as a check that participants were paying attention to the stories, and to ensure they interpreted events appropriately according to the character's perspective (i.e., an inconsistent action from the character should be rated as ‘very inappropriate’). There were eight practice trials to familiarize participants with the procedure, three of which had an example ‘appropriateness’ question after them. The main experimental items were presented in a random order in 10 blocks of 12 stories. Each block was separated by a break, the duration of which was determined by the participant.

#### 2.2.2 Stroop task

A classic Stroop word-based task (Stroop, 1935) was used to measure inhibitory control. Participants were told they would see a word on the computer screen, and were required to respond to the ink colour of the presented word as quickly and as accurately as possible. Responses were made using a button box with four colour buttons – red, green, blue, and yellow. Participants first completed 20 practice trials, including 10 neutral and 10 congruent trials in pseudo-randomised order. For the experimental trials, participants were presented with words printed in red, green, blue, or yellow ink, shown on a grey background. There were 50 congruent trials (e.g., “RED” printed in red ink), 50 incongruent trials (e.g., “RED” printed in blue ink), and 50 neutral trials (i.e., a neutral word such as “MEET”), presented in pseudo-randomised order, in which the same colour word, printed colour, or the same colour word/printed colour pair did not appear on two consecutive trials. A blank screen appeared for 1000  ms at the start of the experimental trials, and the next trial was started immediately after the participant made a response. For analysis, response times for congruent and incongruent trials were log-transformed to adjust for age-related slowing; the dependent variable used for analysis was the Stroop congruency effect (i.e., log-transformed incongruent trial mean response time *minus* log-transformed congruent trial mean response time).

#### 2.2.3 Operation-Span (OSpan)

To assess working memory capacities, the Operation-Span (OSpan; Unsworth, Heitz, Schrock, & Engle, 2005) was used. In the task, participants were asked to solve maths equations whilst concurrently remembering letter sequences. During the task, a maths equation appeared on screen (e.g., (2 × 2)+1 = 3); participants needed to indicate whether the answer presented was correct or incorrect, and there was no time constraint for this response. After the maths equation, a single letter was presented; this was repeated two to seven times (i.e., a minimum of two letters to remember, a maximum of seven letters to remember, in a given trial). Letters were shown for 800 msec. Trials were presented in randomized order, with three trials for each level. This resulted in 18 trials total, including 81 maths problems and 81 letters. At the end of each trial, a 4 × 3 matrix was displayed on the screen, from which participants were required to indicate the letters they had seen, in the correct order, by clicking on a box next to the appropriate letter. There was a ‘blank’ button available to select if participants could not remember the letter in the sequence. There was no time limit for completing this matrix. Participants were given feedback after each trial detailing their word recall accuracy and percentage correct for the maths equations. Participants were encouraged to keep their maths accuracy score at or above 85% throughout the task. The dependent variable used for analysis was a partial OSpan score, calculated as the total number of letters recalled in the correct position.

##### 2.2.3.1 Procedure

The tasks were completed as part of a larger task battery involved in the ‘CogSoCoAGE’ study which lasted approximately 5 h in total. No part of the study procedures or analyses were pre-registered in a time-stamped, institutional registry prior to the research being conducted. Participants completed the tasks over one or two days, and tasks were administered in a counterbalanced order.

### 2.3 EEG recording and analysis

#### 2.3.1 Electrophysiological measures

A Brain Vision Quickamp amplifier system was used with an ActiCap cap for continuous recording of EEG activity from 30 electrodes, referenced to FCz. Vertical electro-oculogram (VEOG) activity was recorded from one extra electrode (below right eye), and horizontal electro-oculogram (HEOG) activity was recorded from one extra electrode (to the left of the left eye). EEG and EOG recordings were sampled at 1000 Hz, and electrode impedance was kept below 10 kΩ. Prior to segmentation offline, a vertical ocular calculation was applied (1*Fp2+(−1*VEOG)). All data were re-referenced to a left/right mastoid average reference. EEG and EOG activity were band-pass filtered (.1–30 Hz, notch filter at 50 Hz). The raw data were visually inspected for noisy sections or channels, and for other general artifacts. EEG activity containing blinks or horizontal eye movements was corrected using a semi-automatic ocular ICA correction approach (Brain Vision Analyzer, 2.1). An average of four ICA components were removed per participant. Semi-automatic artifact detection software (Brain Vision Analyzer 2) was run, to identify and discard trials with non-ocular artifacts (drifts, channel blockings, EEG activity exceeding ± 75 μV). This procedure resulted in an average trial-loss of 10% per condition. Ten participants were removed from analysis due to less than 60% segments remaining in one or more of the critical test conditions after pre-processing, resulting in a final sample size for analysis of 309 participants (101 males).

#### 2.3.2 ERP data analysis

For analysis of scalp-based ERP effects, the signal at each electrode site was averaged separately for each participant and experimental condition, time-locked to the onset of the target word. Waveforms were aligned to a 200 msec baseline prior to target word onset. Mean ERP amplitudes were determined across three time windows (200–400 msec, 400–600 msec, and 600–1000 msec), which allowed us to examine how the detection of belief-inconsistencies evolves over different phases of the N400, and a later ‘wrap-up’ period (reflecting processing at sentence conclusion), and whether effects in these time periods are differentially modulated by age.

To assess ERP amplitudes over lateral electrodes four regions of interests (ROIs) were used, dividing electrodes along a left/right dimension and an anterior/posterior dimension, as in Ferguson, Apperly, et al. (2015) and Ferguson, Cane, et al. (2015). The two ROIs over the left hemisphere were: left-anterior (F7, F3, FC5, FC1), and left-posterior (CP1, CP5, P7, P3, O1); two homologue ROIs were defined for the right hemisphere. ERP amplitudes over midline electrodes (Fz, Cz, Pz, Oz), where the N400 is maximal, were analysed separately from lateral electrode sites. To analyse the N400 in each experimental condition, a repeated-measures ANOVA was performed over lateral electrodes in a 2 (Belief: true *vs* false) × 2 (Consistency: consistent *vs.* inconsistent) × 2 (Hemisphere: left *vs* right) × 2 (Ant/Pos: anterior *vs* posterior) design. For midline electrodes, a 2 (Belief: true *vs.* false) × 2 (Consistency: consistency *vs*. inconsistent) × 4 (Electrode: Fz, Cz, Pz, Oz) repeated-measures ANOVA was used.

## 3 Results

### 3.1 Appropriateness ratings

First, participants’ judgements of appropriateness were analyzed to examine whether participants explicitly detected belief inappropriate behaviors in the stories. A 2 (Belief: true *vs* false) × 2 (Consistency: consistent *vs* inconsistent) repeated-measures ANOVA was run on appropriateness ratings (1 = very appropriate, 5 = very inappropriate).

Both the main effects of Consistency [*F*(1,308) = 941.44, *p* < .001, *ƞ*_*p*_^*2*^ = .75] and Belief [*F*(1,308) = 6.32, *p* = .01, *ƞ*_*p*_^*2*^ = .02] were significant. As expected, participants showed a general tendency to rate belief consistent behaviors lower (i.e., more appropriate) than belief inconsistent behaviors, and rated true-belief stories overall as more appropriate than false-belief stories. Moreover, these main effects were subsumed under an interaction between Belief and Consistency [*F*(1,308) = 94.96, *p* < .001 *ƞ*_*p*_^*2*^ = .24]. This interaction showed that although participants rated consistent behaviors as more appropriate than inconsistent outcomes in both true-belief [*M*_Consistent_ = 1.47; *M*_Inconsistent_ = 3.82; *t*(308) = 34.43, *p* < .001] and false-belief [*M*_Consistent_ = 1.78; *M*_Inconsistent_ = 3.39; *t*(308) = 19.82, *p* < .001] contexts, this difference was larger within true-belief contexts [*t*(308) = 9.75, *p* < .001]*.* Overall, these results indicate that participants successfully tracked the character's beliefs in both true- and false-belief stories, and explicitly detected the false belief, as highlighted by suitable ratings of behavior appropriateness across different contexts.

### 3.2 N400 analyses

**Fig. 1:**
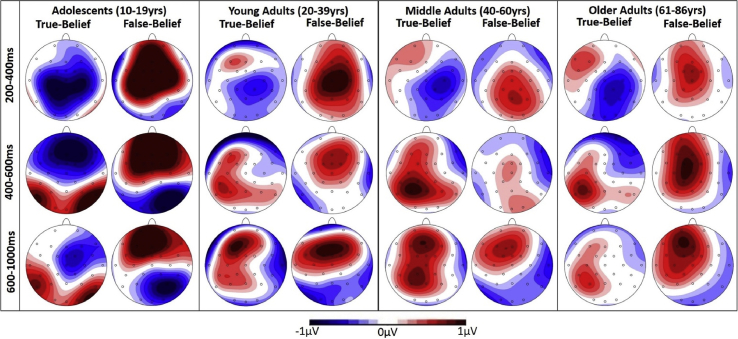
Topographic maps of the ERP difference waveform for each belief context condition (inconsistent *minus* consistent), between 200 and 1000 msec from critical word onset, shown separately for participants in the four age groups described in Table 1 for illustration purposes.

**Fig. 2:**
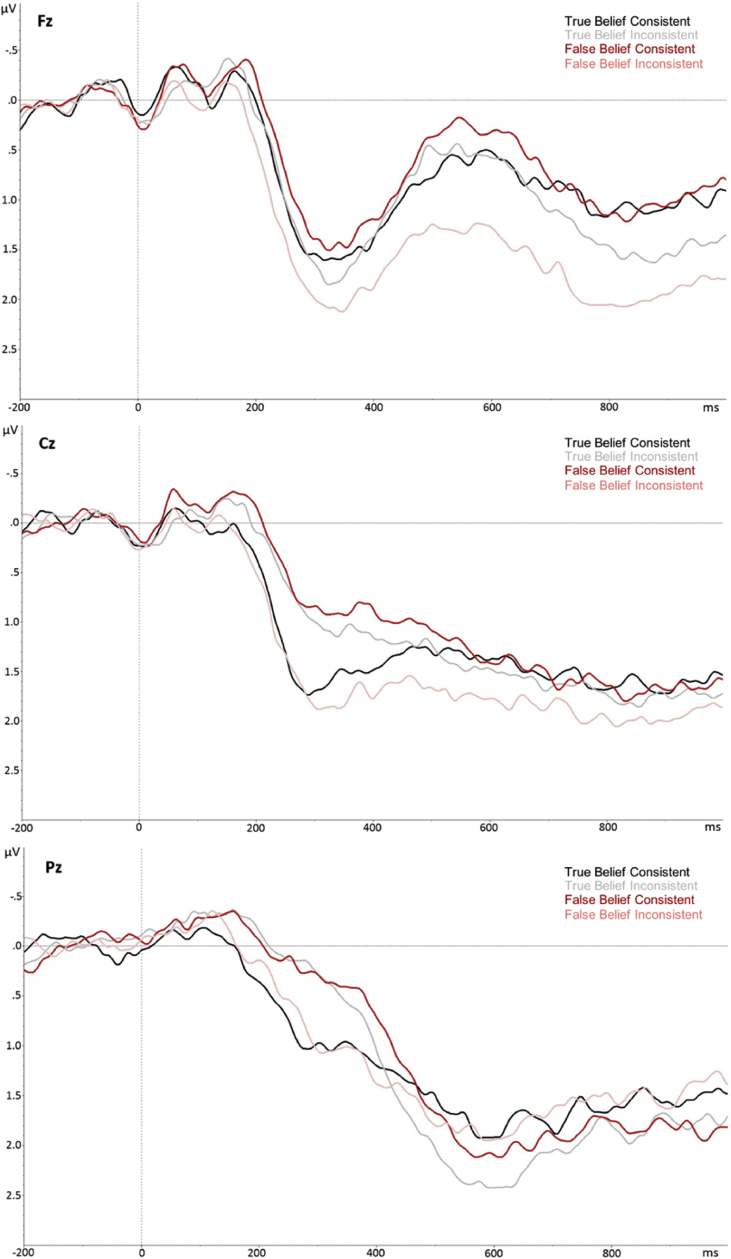
Grand average ERPs over midline electrodes elicited by critical words in the target sentence for each of the four conditions. Note that negativity is plotted upwards.

Full statistical effects for the three time windows (200–400 msec, 400–600 msec, and 600–1000 msec), over lateral and midline electrode sites are summarised in Table 3; full data for each measure is available on the Open Science Framework (https://osf.io/pw7h6/). Note that due to space constraints, only significant effects that involve the Belief or Consistency variables are presented in the text. Fig. 1 shows the topographical maps of ERP difference waveforms for the consistency effect in each belief condition, shown separately for participants in the four age groups described in Table 1 for illustration purposes. Fig. 2 shows the grand average ERP waveforms over three midline electrodes (Fz, Cz, and Pz).

**Table 3:** Results from analyses over lateral and midline electrodes using repeated-measures ANOVAs, in three time windows (200–400 msec, 400–600 msec, 600–1000 msec). Degrees of freedom for all variables = 1,308.

	200–400 msec			400–600 msec			600–1000 msec		
	F	*p*	ƞ_p_^2^	F	*p*	ƞ_p_^2^	F	*p*	ƞ_p_^2^
**Lateral Electrodes**									
Belief	.052	.82	<.001	.055	.814	<.001	.032	.859	<.001
Consistency	.988	.321	.003	1.65	.001***	.033	7.19	.008**	.023
Hemisphere	.409	.523	.001	.007	.935	<.001	.641	.424	.002
Ant/Post	25.84	<.001***	.077	44.14	<.001***	.125	.245	.621	.001
Belief*Consistency	41.18	<.001***	.118	.309	.579	.001	<.001	.991	<.001
Belief*Hemisphere	7.52	.006**	.024	9.29	.003**	.029	2.72	.1	.009
Consistency*Hemisphere	8.35	.004**	.026	16.39	<.001***	.051	29.31	<.001***	.087
Belief*Consistency*Hemisphere	11.9	.001***	.037	2.99	.085	.01	.018	.894	<.001
Belief*Ant/Post	3.26	.072	.01	3.45	.064	.011	3.36	.068	.011
Consistency*Ant/Post	3.25	.073	.01	1.33	.249	.004	7.48	.007**	.024
Belief*Consistency*Ant/Post	2.97	.086	.01	18.22	<.001***	.056	22.88	<.001***	.069
Hemisphere*Ant/Post	16.28	<.001***	.05	9.77	.002**	.031	.934	.335	.003
Belief*Hemisphere*Ant/Post	3.37	.068	.011	3.56	.06	.011	.838	.361	.003
Consistency*Hemisphere*Ant/Post	.978	.323	.003	.821	.366	.003	.613	.434	.002
Belief*Consistency*Hemisphere*Ant/Post	5.38	.021*	.017	1.97	.161	.006	.323	.57	.001
**Midline Electrodes**									
Belief	.033	.856	<.001	.082	.775	<.001	.533	.466	.002
Consistency	2.43	.12	.008	12.6	<.001***	.039	6.07	.014*	.019
Electrode	174.89	<.001	.362	79.06	<.001***	.204	7.74	<.001***	.187
Belief*Consistency	66.87	<.001	.178	3.45	.064	.011	.183	.669	.001
Belief*Electrode	.954	.414	.003	3.53	.015**	.011	3.24	.022*	.01
Consistency*Electrode	1.1	<.001	.032	5.8	.001***	.018	18.76	<.001***	.057
Belief*Consistency*Electrode	8.77	<.001	.031	11.22	<.001***	.035	11.49	<.001***	.036

*Significant at <.05.

**Significant at <.01.

***Significant at <.001.

#### 3.2.1 200–400 msec time window

Analysis of lateral electrodes revealed the expected significant interaction between Belief and Consistency. There were also significant interactions between Belief*Hemisphere, Consistency*Hemisphere, and Belief*Consistency*Hemisphere, which were further subsumed under a four-way interaction between Belief, Consistency, Hemisphere, and Ant/Post. To explore this four-way interaction, post-hoc analyses was conducted using a 2 (Belief) × 2 (Consistency) × 2 (Ant/Pos) repeated-measures ANOVA, separately for the left and right hemispheres (using Bonferroni corrections to control for multiple comparisons, leading to a new significance criterion of .025). For the left hemisphere, results showed a significant Belief*Consistency interaction [*F*(1,308) = 11.46, *p* = .001, *ƞ*_*p*_^*2*^ = .036], which was further subsumed under a three-way interaction with Anterior/Posterior [*F*(1,308) = 5.84, *p* = .016, *ƞ*_*p*_^*2*^ = .019]. This three-way interaction reflected a significant Belief*Consistency interaction in left-posterior regions [*F*(1,308) = 18.42, *p* < .001, *ƞ*_*p*_^*2*^ = .056], which was not present in left-anterior regions [*F*(1,308) = .671, *p* = .413, *ƞ*_*p*_^*2*^ = .002]. For the right hemisphere, there was a significant Belief*Consistency interaction [*F*(1,308) = 60.16, *p* < .001, *ƞ*_*p*_^*2*^ = .163], but no three-way interaction with Anterior/Posterior [*p* = .385]. Where this Belief*Consistency interaction was significant, it reflected the classic N400 for true-belief stories (i.e., a more negative-going wave-form for inconsistent *vs* consistent; all *t*s > 2.98, *p*s < .003), but a reversed N400 effect for false-belief stories (i.e., a more negative-going wave-form for consistent *vs* inconsistent; all *t*s > 3.23, *p*s < .005).

Over midline electrodes, there were significant interactions between Belief*Consistency, and Consistency*Electrodes, both of which were further subsumed under a three-way interaction between Belief, Consistency, and Electrode. Post-hoc analyses, using Bonferroni corrections to control for multiple comparisons, leading to a new significance criterion of .0125, revealed that the Belief*Consistency interaction was significant at Fz [*F*(1,308) = 15.11, *p* < .001, *ƞ*_*p*_^*2*^ = .047], Cz [*F*(1,308) = 74.38, *p* < .001, *ƞ*_*p*_^*2*^ = .20], and Pz [*F*(1,308) = 43.88, *p* < .001, *ƞ*_*p*_^*2*^ = .13], but not at Oz [*F*(1,308) = 5.37, *p* = .021, *ƞ*_*p*_^*2*^ = .02]). Over electrodes Cz and Pz, the interaction showed that the N400 was more negative-going for inconsistent *vs* consistent in true-belief stories (*t*s > 4.87, *p*s < .001), but more negative-going for consistent versus inconsistent in false-belief stories (*t*s > 4.65 *p*s < .001). At Fz, the consistency effect was only significant for false-belief stories [*t*(308) = 5.66, *p* < .001; consistent < inconsistent].

In summary, results in the 200–400 msec time window supported our first hypothesis, showing a typical N400 effect in true-belief conditions (i.e., more negative-going waveforms for inconsistent *vs* consistent story outcomes), and a reversed N400 effect in false-belief conditions (i.e., more negative-going waveforms for consistent *vs* inconsistent story outcomes), indicating egocentric processing of the stories in false-belief scenarios.

#### 3.2.2 400–600 msec time window

Analysis of lateral electrodes revealed a significant main effect of Consistency, which was further modulated by Hemisphere. This Consistency*Hemisphere interaction revealed more negative-going N400 amplitudes for consistent versus inconsistent conditions in the left hemisphere [*t*(308) = 4.85, *p* < .001], but no significant difference in the right hemisphere [*t*(308) = .60, *p* = .55]. There was also a significant interaction between Belief and Hemisphere, however, none of the post-hoc comparisons reached significance (all *t*s < 1.7). More importantly, the three-way interaction between Belief, Consistency, and Ant/Post was significant. Post-hoc analyses using Bonferroni corrections to control for multiple comparisons, leading to a new significance criterion of .025, showed that the Belief*Consistency interaction was significant for both anterior [*F*(1,308) = 12.48, *p* < .001, *ƞ*_*p*_^*2*^ = .04] and posterior electrodes [*F*(1,308) = 8.26, *p* = .004, *ƞ*_*p*_^*2*^ = .03], reflecting different patterns in each region. Over anterior electrodes, the N400 was more negative-going for consistent versus inconsistent in false-belief stories [*t*(308) = 3.59, *p* < .001], but did not differ in true-belief stories [*t*(308) = 1.73, *p* = .084]. Over posterior electrodes, the N400 was more negative-going for consistent versus inconsistent in true-belief stories [*t*(308) = 4.21, *p* < .001], but did not differ in false-belief stories [*t*(308) = .17, *p* = .867].

The midline electrode analysis showed a significant main effect of Consistency (consistent < inconsistent), as well as significant interaction effects between Belief*Electrode and Consistency*Electrode, which were further subsumed in a three-way interaction between Belief, Consistency, and Electrode. Post-hoc analyses, using Bonferroni corrections to control for multiple comparisons, leading to a new significance criterion of .0125, revealed that the Belief*Consistency interaction was significant at electrodes Fz [*F*(1,308) = 21.19, *p* < .001, *ƞ*_*p*_^*2*^ = .06] and Cz [*F*(1,308) = 9.46, *p* = .002, *ƞ*_*p*_^*2*^ = .03], but not at electrodes Pz [*F* = 2.36, *p* = .13] and Oz [*F* = 1.55, *p* = .21]. Over electrodes Fz and Cz, the interaction showed that the N400 was more negative-going for consistent versus inconsistent in false-belief stories (*t*s > 4.61, *p*s < .001), but did not differ in true-belief stories (*t*s < .72 *p*s > .47).

In summary, results in the 400–600 msec time window showed a reversed N400 effect in false-belief conditions across anterior electrodes (i.e., more negative-going waveforms for consistent *vs* inconsistent story outcomes), indicating egocentric processing of the stories in false-belief scenarios. In true-belief contexts, the typical N400 effect was now reversed so that the N400 was more negative-going for consistent versus inconsistent stories over posterior electrodes.

#### 3.2.3 600–1000 msec time window

Analysis of lateral electrodes revealed a significant main effect of Consistency, which was further modulated by Hemisphere. This Consistency*Hemisphere interaction revealed more negative-going N400 amplitudes for consistent versus inconsistent conditions in the left hemisphere [*t*(308) = −4.90, *p* < .001], but no significant difference in the right hemisphere [*t*(308) = .359, *p* = .72]. There was also a significant interaction between Consistency and Ant/Post; this interaction reflected more negative-going amplitudes for consistent versus inconsistent conditions in anterior regions [*t*(308) = −3.52, *p* < .001], but no significant difference in the posterior regions [*t*(308) = −.157, *p* = .876]. More importantly, the three-way interaction between Belief, Consistency, and Ant/Post was significant. Post-hoc analyses using Bonferroni corrections to control for multiple comparisons, leading to a new significance criterion of .025, showed that the Belief*Consistency interaction was significant for both anterior [*F*(1,308) = 8.46, *p* = .004, *ƞ*_*p*_^*2*^ = .027] and posterior electrodes [*F*(1,308) = 8.93, *p* = .003, *ƞ*_*p*_^*2*^ = .028], reflecting different patterns in each region. Over anterior electrodes, the N400 was more negative-going for consistent versus inconsistent in false-belief stories [*t*(308) = −4.65, *p* < .001], but did not differ in true-belief stories [*t*(308) = −.613, *p* = .541]. Over posterior electrodes, the N400 was more negative-going for consistent versus inconsistent in true-belief stories [*t*(308) = −2.21, *p* = .028], but was more negative-going for inconsistent versus consistent in false-belief stories [*t*(308) = 2.17, *p* = .031].

Midline electrode analysis showed a significant main effect of Consistency (consistent < inconsistent), as well as significant interaction effects between Belief*Electrode and Consistency*Electrode, which were further subsumed in a three-way interaction between Belief, Consistency, and Electrode. Post-hoc analyses, using Bonferroni corrections to control for multiple comparisons, leading to a new significance criterion of .0125, revealed that the Belief*Consistency interaction was significant at electrodes Fz [*F*(1,308) = 9.82, *p* = .002, *ƞ*_*p*_^*2*^ = .031] and Pz [*F*(1,308) = 8.95, *p* = .003, *ƞ*_*p*_^*2*^ = .028], but not at electrodes Cz [*F*(1, 308) = 1.01, *p* = .316, *ƞ*_*p*_^*2*^ = .003] or Oz [*F*(1,308) = 4.27, *p* = .040, *ƞ*_*p*_^*2*^ = .014]. Over electrode Fz, the interaction showed that the N400 was more negative-going for consistent versus inconsistent in false-belief stories [*t*(308) = −6.33, *p* < .001], with no significant difference in true-belief stories [*t*(308) = −2.44, *p* = .015]. Over electrode Pz, there was no significant difference in N400 amplitudes in either true-belief [*t*(308) = −2.25, *p* = .025] or false-belief [*t*(308) = 2.23, *p* = .027] stories.

In summary, results in the 600–1000 msec time window showed a reversed N400 effect in false-belief conditions across anterior electrodes (i.e., more negative-going waveforms for consistent *vs* inconsistent story outcomes), indicating continued egocentric processing of the false-belief scenarios. In true-belief stories, the N400 was again more negative-going for consistent versus inconsistent stories across posterior electrodes.

### 3.3 Correlations

Next, we investigated whether and how age influenced the detection of story character's inconsistent behaviors, within true-belief and false-belief contexts.^2^ In particular, we aimed to examine whether the initial egocentric interpretation of story events (i.e., the reversed N400 effect within false-belief stories) was driven by, or enhanced in, the older participants in our sample, reflecting an age-related decline in ToM ability. In addition, given the robust evidence for age-related decline in cognitive skills that have been associated with false belief reasoning (i.e., working memory and inhibitory control), we examined whether individual differences in these skills predicted the detection of inconsistencies in the story comprehension task.

**Fig. 3:**
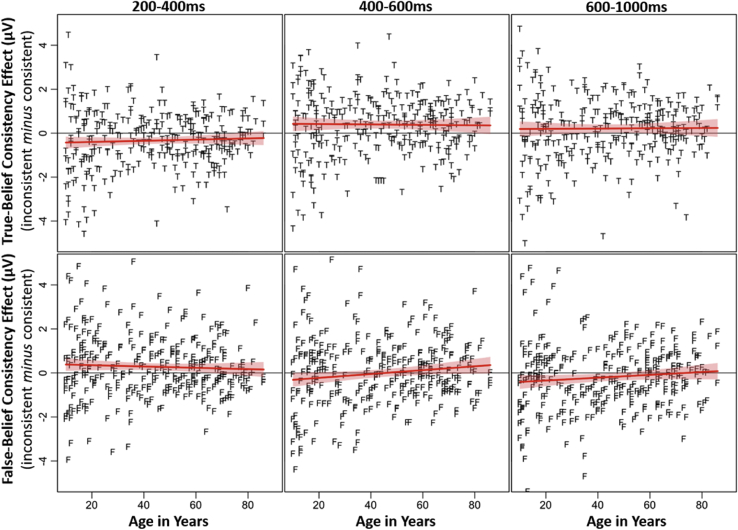
Correlations between age (years) and the N400 consistency effect (inconsistent *minus* consistent), plotted separately for ERP effects in the three time windows (200–400 msec, 400–600 msec, and 600–1000 msec) across posterior regions. Data for true-belief stories are shown with the letter ‘T’ in the upper panels, and data for false-belief stories are shown with the letter ‘F’ in the lower panels.

To facilitate these analyses, a ‘consistency effect’ variable was calculated for each individual, separately for true-belief and false-belief conditions. N400 amplitudes were pooled over posterior electrode sites (CP5, CP1, CP2, CP6, P7, P3, P4, P8, O1, O2), and difference scores were calculated by subtracting the mean N400 amplitude in the consistent condition from the inconsistent condition, separately for the three time-windows (200–400 msec, 400–600 msec, 600–1000 msec). Here, a negative score indicates a larger N400 effect for the inconsistent compared to consistent condition (i.e., appropriate anomaly detection), and a more positive score indicates a larger effect for the consistent compared to inconsistent condition (i.e., interpreting events egocentrically in false belief contexts). Individual differences in working memory capacity and inhibitory control were assessed using the partial OSpan score and the Stroop congruency effect, respectively. Note that due to computer error or participant refusal to complete task, data was missing for eight participants on the OSpan task, and three from the Stroop task. Full statistical effects for these correlation analyses are presented in Table 4. Fig. 3 shows correlations with age and the consistency effect in the three N400 time windows.

**Table 4:** Correlations (*N* = 309) between age and the N400 consistency effect (posterior region) in three time-windows (200–400 msec, 400–600 msec, and 600–1000 msec), across true/false belief contents, and executive function abilities (working memory [Ospan] and inhibition [Stroop]).

	Age	200–400 msec		400–600 msec		600–1000 msec		Working Memory	Inhibition
		True Belief	False Belief	True Belief	False Belief	True Belief	False Belief		
**Posterior Electrodes**									
**Age**	–								
**200**–**400 msec**									
True Belief	.045	–							
False Belief	−.047	−.026	–						
**400**–**600 msec**									
True Belief	−.013	.649***	.015	**-**					
False Belief	.123*	−.059	.635***	−.202***	–				
**600**–**1000 msec**									
True Belief	.008	.487***	.001	.793***	−.153**	–			
False Belief	.091	.021	.581***	−.086	.720***	−.074	–		
**Working Memory**	−.314***	−.025	.050	.058	−.026	.017	−.026	–	
**Inhibition**	−.203***	−.089	.071	−.023	.037	−.044	.045	.181**	–

*Significant at < .05.

**Significant at < .01.

***Significant at < .001.

Analysis of the posterior electrodes showed that in the 200–400 msec N400 time window, age did not significantly correlate with the inconsistency effect in either true-belief or false-belief contexts. Importantly, this suggests that across all ages, participants initially anchored comprehension of false-belief stories to their own egocentric perspective rather than to the character's more limited knowledge, and that this egocentric tendency did not increase with advancing age. In the 400–600 msec time window, age did not significantly correlate with the consistency effect in true-belief trials, however results revealed a significant positive correlation between age and the consistency effect in false-belief trials^3^ [*r*(309) = .123, *p* = .03]. Here, the size of the consistency effect increased with increasing age, which suggests that false-belief events were more likely to be interpreted from an egocentric perspective in older age. For the 600–1000 msec time window, there were no significant correlations with age for the consistency effect in either true- or false-belief trials.

Finally, as expected, age was significantly negatively correlated with both working memory capacity [*r*(301) = −.314, *p* < .001] and inhibitory control [*r*(306) = −.203, *p* < .001], replicating the robust patterns of age-related cognitive decline seen in previous research among our sample. Importantly, neither of these cognitive skills significantly correlated with N400 responses.

## 4 Discussion

The ability to understand another person's perspective is an important part of daily life, requiring rapid and efficient computation of how another person's perspective may differ from one's own current perspective. In this paper, ERP measures were used to assess implicit ToM abilities in a belief-state manipulation task. We examined effects of expectancy during narrative comprehension on the established N400 effect, comparing neural responses to belief-consistent versus belief-inconsistent outcomes in true and false belief contexts. The key aim of the study was to examine whether and how implicit ToM changes across the lifespan (i.e., whether the extent of ‘egocentric bias’ changes across different ages, or whether it remains stable from adolescence through to older age). A unique feature of our study is the large sample (N = 309) of individuals, aged between 10 and 86 years old, which allowed us to track changes across an extended period of development. In addition, we explored whether any age-related changes in ToM capacities can be explained by domain-general changes in cognitive abilities (specifically working memory and inhibitory control) as opposed to domain-specific changes in social-cognitive abilities.

Our results first established that people can successfully track a story character's beliefs in true- and false-belief contexts (i.e., correctly rating story outcomes as appropriate *vs* inappropriate), indicating explicit understanding of false-belief scenarios across the lifespan. Following this, we examined implicit belief processing by analyzing N400 responses to different task conditions (belief-type and consistency) across the full sample to establish whether participants were sensitive to violations of expectation from the perspective of a story character, or were biased to their own situational knowledge (i.e., how egocentric was the processing of the stories). This analysis was conducted across three time windows – 200–400 msec, 400–600 msec, and 600–1000 msec – to allow examination of the timings of these effects and whether effects in these time periods are modulated by age. Finally, an N400 consistency effect was calculated for true- and false-belief contexts separately, to assess whether individual differences among participants – specifically, inhibitory control and working memory abilities – modulates their ability to successfully consider another person's perspective, and how this ability changes with age.

Across all participants, results showed that when a story character was in possession of a true-belief about an object's location, the N400 was more negative-going for belief-*inconsistent* than for belief-consistent outcomes, as is typical for semantic and discourse anomalies Camblin et al., 2007, Nieuwland et al., 2019, Van Berkum et al., 1999. In the true-belief context, this pattern was attenuated or reversed in later time windows, likely reflecting a tendency for participants to reprocess the critical word given the uncertain context of the experiment (i.e., ~33% of items included a violation). Critically, and in line with our first hypothesis, overall analysis of false-belief trials showed the opposite pattern of results. That is, in false-belief contexts, belief-*consistent* outcomes led to more negative-going N400 effects than belief-inconsistent outcomes, suggesting that participants were processing the stories from an egocentric perspective. These results replicate the pattern seen in Ferguson, Apperly, et al. (2015) and Ferguson, Cane, et al. (2015) and indicate that when listening to the stories, participants do not immediately integrate the character's beliefs, but instead initially anchor understanding to their own more informed knowledge of the world. These findings fit with previous research that has indicated that when engaging ToM capacities, events are often processed using an initial egocentric bias (e.g., Birch and Bloom, 2007, Bradford et al., 2018, Ferguson and Breheny, 2012, Ferguson, Apperly and Ahmad et al., 2015, Keysar et al., 2000), despite explicit measures of ToM (such as the appropriateness ratings in the current task) highlighting that participants are able to ultimately overcome this initial bias, and successfully consider the wider context of the story to identify whether a character's actions made sense or not.

This study makes further important contributions to the literature by examining how implicit ToM abilities mature and develop across the lifespan, from adolescence to healthy old age. Correlation analyses revealed no significant relationship between age and the consistency effect in the early time window (200–400 msec after stimulus onset) for either true- or false-belief conditions, indicating that during this period, all participants – regardless of age – showed an egocentric bias in processing of the stories presented. This is important, because it shows that egocentric processing is the default perspective for information integration, and that this spontaneous ‘pull of reality’ effect does not increase with advancing age (i.e., goes against the suggestion that belief processing gets worse in older age). However, in the 400–600 msec time window, age was found to influence the magnitude of the consistency effect in false-belief (but not true-belief) contexts; as age increased the N400 consistency effect also increased. Although this effect was relatively weak (*r* = .123) and thus requires further research to more firmly establish the magnitude of this finding, the result provides preliminary evidence that the tendency to interpret story events egocentrically, rather than according to the story character's more limited beliefs, increases with advancing age. In other words, older adults' neural activity continues to reflect an interpretation of the story events according to their own knowledge of reality rather than the character's false belief (i.e., a reversed N400, reflecting more negative-going N400 for consistent *vs* inconsistent), while younger participants had now switched to accommodate the character's actions according to that character's own (false) belief state (i.e., a classic N400, reflecting more negative-going N400 for inconsistent *vs* consistent). By the 600–1000 msec ‘wrap-up’ window, this age modulation was no longer present. We interpret these results cautiously, given the magnitude of the correlation in the 400–600 msec time window, and the challenge of linking cognitive outcomes to that of neural data. Taken together, however, the results tentatively suggest that whilst all participants initially processed events in the stories egocentrically, anchoring comprehension to the ‘self’ perspective, younger participants more quickly switched to integrate events according to the story character's perspective compared to older adults, who continued to experience interference from their own egocentric perspective until later in the trials.

It is noted that, in using a cross-sectional design, such as the current study, it is difficult to ascertain to what extent these changes are due to age-related differences – for this, longitudinal analysis would be required, assessing participants across different time points. However, within the scope of this study design, our data provides novel evidence that social cognitive skills do undergo changes with advancing age, although even younger participants experience some initial difficulty adopting others’ mental states, originally processing the stories from an egocentric perspective. Future research using a longitudinal sample would be valuable for teasing apart the role of ageing, within-person changes, as opposed to cross-sectional differences (e.g., Lindenberger, von Oertzen, Ghisletta, & Hertzog, 2011).

It is important to note that we have interpreted our ERP effects within the constraints of the N400 component, and the established cognitive processes that modulate it, since our a-priori predictions were based on this. However, we note that the topography of some of our effects is uncharacteristic of typical N400 effects (i.e., the consistency effect in false-belief contexts was maximal over fronto-central rather than centro-parietal scalp sites). It is possible that this unusual pattern was driven by the auditory modality in which the true/false belief stories were presented (it has been shown that auditory language elicits a slightly more frontal N400 than language presented in the visual modality; Kutas and Federmeier, 2011, Kutas and Van Petten, 1994). Alternatively, it could reflect a different component all together. Specifically, prior research has observed a later occurring ‘semantic P600’ component that is elicited when participants are detecting ‘semantic reversal anomalies’, or pragmatic violations and ambiguities (e.g., Kim and Osterhout, 2005, Osterhout and Holcomb, 1992, Schacht et al., 2014). The P600 has been suggested to play a role in indicating a need for reanalysis following a language/lexical violation in a sentence (e.g., Kuperberg et al., 2003, Münte et al., 1998). Within this definition of the semantic P600, it is possible that the ‘reversed N400’ seen in later time windows for the true-belief context and all time windows for the false-belief context (i.e., more positive-going waveform for inconsistent *vs* consistent critical words) reflects greater reanalysis of the input following a belief-inconsistent word compared to a belief-inconsistent word (since listeners reintegrate information after detecting the violation), and thus reflects appropriate language processing and reinterpretation in the false-belief context (i.e., reanalysis to fit with the character's false belief). This interpretation fits with the explicit ratings of appropriateness, which showed that participants successfully tracked the character's true and false beliefs by the end of the story. Moreover, if the correlation effects in the later ‘N400’ time period were interpreted as a semantic P600, this would indicate that older adults showed a *greater* reanalysis for false-belief inconsistencies than younger participants, indicating a processing advantage for older adults who are more likely to re-analyse and ‘repair’ sentences in order to make sense of them. Distinguishing these two components using source analysis on the current data is beyond the scope of this paper, but future research may focus on examining the role of age in predicting N400 and semantic P600 components separately in language processing tasks.

Our third question asked whether any age-related changes in ToM can be attributed to domain-general changes, or whether they are specific to social abilities. Correlation analysis highlighted a negative correlation between age and both inhibitory control and working memory, indicating that these abilities decline with age, as expected (Braver and West, 2008, De Luca et al., 2003, Salthouse, 2009, Schroeder and Salthouse, 2004). Both of these executive skills are argued to play an important role in ToM engagement – inhibitory control allowing suppression of one's own perspective in favor of another person's, and working memory allowing multiple mental states to be represented simultaneously. Importantly, the current results highlighted that, contrary to our third hypothesis and despite these age-related declines in executive functions, age effects seen in the N400 consistency effect for false-belief trials in the 400–600 msec time window were not reflective of a domain-general decline (due to the absence of correlations with either executive function ability), but rather a more domain-specific decline in implicit social-cognitive processing abilities. In fact, this lack of domain-general input from cognitive abilities concords with recent data from Martin et al. (2019) showing that an age-related decline in visual perspective-taking (specifically switching from the self to other perspective) is only partially mediated by declines in inhibitory control (and not at all by working memory). In our study, this is further supported by the correlation with age being restricted to false-belief conditions, rather than true-belief conditions, indicating that declines in abilities as a result of increasing age are specifically related to an individual's ability to infer and use ToM online. In true-belief conditions, there is no conflict between the participant's and story character's belief states, which are both aligned with reality, thus removing any potential interference effects between the two different perspectives. These data suggest that age-related declines in ToM are specific to the social domain, although it remains to be ascertained to what extent the changes in ERP amplitudes established in the current study relate to explicit social cognition performance, and thus the extent to which ageing effects influence engagement in social cognition capacities in everyday life.

Interestingly, the results from the current study were established despite the older adult group having, on average, an IQ over 10 points higher than other groups, reflecting the high-functioning cognition of the older participants in this sample. To further examine the role of age in predicting differences in the N400 effect, it may be of interest in further research to examine how individuals with higher versus lower IQ within one age category differentially perform on behavioural and neural measures of ToM, establishing whether IQ influences age-related changes in ToM performance. Additionally, the adolescent group in the current study showed considerable variation in performance, as highlighted in Fig. 3. The presence of this variation supports prior research that has suggested large individual differences in neurodevelopmental trajectories across individuals during adolescence (e.g., Dumontheil, 2016, Foulkes and Blakemore, 2018, Tamnes et al., 2018), and highlights the importance of including adolescents in research examining the development of ToM into adulthood, with significant changes present throughout adolescence. This critical period of social, psychological, and biological development (Foulkes & Blakemore, 2018) would be interesting to examine in more refined detail than permitted by the scope of the current paper, exploring how ToM processing may change across the adolescent period, and how individual differences may influence these changes.

In sum, the results of the current study make novel contributions to the field by demonstrating that, although older adults were able to explicitly evaluate story events according to a character's (false) beliefs, they experience age-related changes at an implicit level. Older participants maintained an egocentric stance for longer than younger participants, who were able to switch to take the character's more limited perspective at a later stage of online comprehension. Prior research has suggested a differentiation between explicit and implicit ToM abilities, with older adults experiencing greater difficulties than younger adults when engaging in explicit ToM tasks (e.g., Bailey and Henry, 2009, German and Hehman, 2006, Grainger et al., 2015, Maylor et al., 2002), but showing less age-related changes in tasks assessing implicit ToM, where older and younger adults perform comparably (e.g., Grainger et al., 2018). In the current study, the need to draw ToM inferences was not strictly implicit in the task (i.e., participants were asked to judge the appropriateness of the character's actions after some sentences, thus were cued to attend to the character's perspective), but our ERP data highlights age-related changes in implicit measures, indicating a possible explanation for experiences of declining social skills at older ages. The results highlight that with increasing age, interference from the self perspective is more pervasive, and inhibition of this egocentric perspective takes longer to achieve. In turn, this could lead to repercussions during social interactions, with delayed processing of the perspectives of other people when engaging in social situations. Further research is needed to establish the impact that this elongated egocentric bias in online ToM may have on everyday social functioning.

## 5 Conclusion

In conclusion, the results of the current study provide novel evidence that people experience an egocentric bias in implicit ToM processing that persists throughout the lifespan. When listening to stories in which a character is described as having a true-belief about an objects location, a standard N400 effect is elicited when the character acts in an inconsistent manner (*vs* consistent). In contrast, in false-belief conditions an enhanced N400 effect is seen when a character acts in a belief-*consistent* manner (*vs* belief-inconsistent), indicating that the stories have been processed from the participant's own perspective, failing to spontaneously consider the perspective of the character. ERP results showed that in an early time window (200–400 msec post stimulus onset) there was no significant effect of age, suggesting that all individuals demonstrated this egocentric effect to a comparable degree. In contrast, a significant effect of age emerged in the later time window (400–600 msec), suggesting that increasing age led to a longer time taken to disengage from the self-perspective to consider the character's perspective. In the final 600–1000 msec time window, this age modulation was no longer present. Importantly, these age-related changes were not as a result of domain-general cognitive changes, with no significant relationship to executive function abilities, suggesting a more domain-specific social-cognitive decline in online ToM. This study demonstrates the importance of examining implicit ToM processes in healthy aging populations – including both adolescence, which is a period of rapid developmental change, and older aged adults – allowing further understanding of how ToM functions and develops across the lifespan to be established.

## Open practices

The study in this article earned Open Materials and Open Data badges for transparent practices. Materials and data for the study are available at https://osf.io/pw7h6/.

## Footnotes

2 Please see Supplementary Materials on OSF (https://osf.io/pw7h6/?view_only=9bb79f149f02419285ccaf8c8b89caf3) for details of the main ANOVA analyses run separately for the four age groups described in Table 1.

3 Note that follow-up stepwise multiple regression analysis showed that the linear model best described the data [*F*(1,307) = 4.75, *p* = .030], and adding the quadratic coefficient (that might best fit a pattern where belief reasoning skills continue to develop through adolescence, reaching a peak at some point in adulthood, before declining in older age) did not significantly change the R^2^ from the linear model [*F*(1,306) = 2.56, *p* = .11].
